# An Extracellular Matrix Overlay Model for Bioluminescence Microscopy to Measure Single-Cell Heterogeneous Responses to Antiandrogens in Prostate Cancer Cells

**DOI:** 10.3390/bios14040175

**Published:** 2024-04-05

**Authors:** Audrey Champagne, Imene Chebra, Pallavi Jain, Cassandra Ringuette Goulet, Annie Lauzier, Antoine Guyon, Bertrand Neveu, Frédéric Pouliot

**Affiliations:** 1 Centre de Recherche du CHU de Québec, Université Laval, Quebec, QC G1V 4G2, Canadaimen.chebra@crchudequebec.ulaval.ca (I.C.); pallavi.jain87@gmail.com (P.J.); cassandra.r.goulet@gmail.com (C.R.G.); annie.lauzier@crchudequebec.ulaval.ca (A.L.); antoine.guyon@crchudequebec.ulaval.ca (A.G.);; 2 Department of Surgery (Urology), Faculty of Medicine, Laval University, Quebec, QC G1R 2J6, Canada

**Keywords:** single cell, drug response, transcriptional-based biosensor, bioluminescence imaging, Matrigel, extracellular matrix, PSA, antiandrogen, prostate cancer, heterogeneity

## Abstract

Prostate cancer (PCa) displays diverse intra-tumoral traits, impacting its progression and treatment outcomes. This study aimed to refine PCa cell culture conditions for dynamic monitoring of androgen receptor (AR) activity at the single-cell level. We introduced an extracellular matrix-Matrigel (ECM-M) culture model, enhancing cellular tracking during bioluminescence single-cell imaging while improving cell viability. ECM-M notably tripled the traceability of poorly adherent PCa cells, facilitating robust single-cell tracking, without impeding substrate permeability or AR response. This model effectively monitored AR modulation by antiandrogens across various PCa cell lines. Single-cell imaging unveiled heterogeneous antiandrogen responses within populations, correlating non-responsive cell proportions with drug IC50 values. Integrating ECM-M culture with the PSEBC-TSTA biosensor enabled precise characterization of ARi responsiveness within diverse cell populations. Our ECM-M model stands as a promising tool to assess heterogeneous single-cell treatment responses in cancer, offering insights to link drug responses to intracellular signaling dynamics. This approach enhances our comprehension of the nuanced and dynamic nature of PCa treatment responses.

## 1. Introduction

It is acknowledged that human cancers harbor substantial intra- and inter-tumoral cell heterogeneity. Tumors are, hence, composed of sub-populations of cells with distinct genetic and phenotypic features, influencing disease progression, metastasis, and treatment response [1,2,3]. The selection of effective therapies requires a better understanding of cancer cell heterogeneity. Biosensors that monitor intra-tumoral heterogeneity are, therefore, relevant and better than bulk tissue biomarkers to guide patient care and therapeutic decisions [4,5,6,7,8].

Prostate cancer (PCa) is the most frequently diagnosed non-skin cancer and is a significant cause of cancer-related death in North American men [9]. PCa growth is mainly driven by the androgen receptor (AR) transcriptional activity, and androgen deprivation therapy (ADT—also called castration) is the backbone of PCa systemic therapy. Despite initial response to ADT in a vast majority, all patients will progress to castration-resistant prostate cancer (CRPC) when undergoing palliative ADT. In the last five years, the antiandrogen arsenal to treat CRPC has dramatically increased with the introduction of new AR-axis-targeted therapies (ARAT), such as enzalutamide (Enza), apalutamide (Apa) and darolutamide (Daro) [10], which complement the first-generation anti-androgen Bicalutamide (Bica). These second-generation androgen receptor inhibitors (ARi) have a 7- to 10-fold increased affinity for androgen receptors [11,12,13]. They bind directly to the active site of the androgen receptor, impeding its nuclear translocation, inhibiting DNA binding, and, consequently, reducing androgen receptor-mediated transcription and cancer growth/progression [14,15]. Although CRPC is lethal, Enza, Apa and Daro have significantly improved metastasis-free and overall survival in advanced PCa patients undergoing palliative ADT [10]. The molecular mechanisms driving advanced PCa progression to CRPC are numerous. In addition to AR-dependent resistance mechanisms, such as AR mutations, splice variants and amplification [16,17,18,19], many AR independent mechanisms have been described. PTEN/PI-3 kinase, DNA repair, Wnt and cell cycle pathways are altered in 71, 49, 23, 18, >21% of patients, respectively [20,21], and 15 to 20% of PCa patients under hormonal therapies show histological evidence of neuroendocrine prostate cancer (NEPC) cell differentiation or AR-indifference state [22,23,24,25]. Molecular markers that help characterize sub-population heterogeneity in PCa and guide clinical decision making in concordance with the evolving array of potential treatments would be invaluable. Although biomarkers have been validated for clinical use in PCa detection, none of them address the complex antiandrogen response heterogeneity [21,26,27,28,29,30].

We previously developed a bioluminescence microscopy method based on a specific biosensor, PSEBC-TSTA, that can dynamically monitor single-cell AR transcriptional activity via bioluminescence microscopy [31,32,33,34,35]. Our system relies on the transcriptional activity of the prostate-specific antigen (PSA) gene promoter (PSEBC) to detect AR activity with high specificity and sensitivity in PCa cells [31,32]. We have shown that our biosensor could discriminate the cell population’s overall ARi sensitivity. However, the poor adherence and/or motility of PCa cells make single-cell tracking over the course of antiandrogen treatment challenging, limiting the potential of this method for the characterization of the heterogenous antiandrogen response at the single-cell level in the tumor cell population.

To effectively track cells using bioluminescence over time, a protocol to immobilize isolated cells that is compatible with bioluminescent imaging modality needs to be developed. The technique used should not hamper cell viability and allow unhindered supply of D-luciferin substrate to the cells. While surface adsorption techniques have limited utility, with very low or non-adherent cells, such as PCa cells, the matrices used in trapping techniques help maintain cell viability and provide a 3D environment for cellular growth and proliferation [36,37]. These matrices can be inert, like calcium alginate, agarose or chitosan. Even though they provide a solid inert support to mimic a tissue-like structure, these matrices often do not participate in signal transduction and can result in signal loss or background noise. Extracellular matrices (ECMs) like Matrigel (ECM-M), on the other hand, are rich in extracellular components and contain several growth factors [38]. ECM-M is widely used for organoid culture, owing to its molecular and structural properties that favor cell adhesion, proliferation and viability, and, more specifically, in prostate cancer organoids [39]. Herein, we present a groundbreaking ECM-M culture model, designed for investigating the single-cell therapeutic response in prostate cancer (PCa) using bioluminescence microscopy. This innovative model involves the application of ECM-M directly on top of the cells, a departure from the conventional method of ECM-M coverage across the entire culture plate. Through meticulous optimization of ECM-M density to enhance cell immobilization, we have pioneered a novel approach that allows for precise tracking of single cells. Our study focuses on exposing different PCa cell lines to ARi therapies and evaluating their responses under conditions with or without ECM-M. By showcasing the effectiveness of the ECM-M overlay in accurately tracking poorly adherent cells, we established a robust platform for determining cellular sensitivity to antiandrogens using dynamic bioluminescence microscopy at the single-cell level.

## 2. Materials and Methods

### 2.1. Plasmid Construction and Adenoviral Production

Adenoviral plasmids for PSEBC-TSTA and CMV-TSTA were previously described [31]. Lentivirus-expressing Renilla luciferase and GFP used in LNCaP cells were constructed using the plasmid pccl-CMV-RL-IRES-EGFP [33]. Adenoviral backbone plasmids were transfected into 293A cells for viral production. Amplified virus particles were column purified using Adeno-X™ Maxi purification kit (Takara Bio USA, Mountain View, CA, USA) and stored in buffer A195 after buffer exchange [40]. Titration of each virus was performed using Adeno-X™ Rapid Titer Kit (Takara).

### 2.2. Cell Line Culture

For this study, we selected less adherent and non-adherent prostate cancer (PCa) cell lines LNCaP and LAPC9 to assess the impact of ECM-M on cell tracking [41]. However, to evaluate the sensitivity of PCa cell lines to antiandrogens using bioluminescence microscopy, we specifically chose the enzalutamide-sensitive PCa cell lines (LNCaP and LAPC4), as well as the non-sensitive PCa cell line 22Rv1, as a control model for the PSEBC-TSTA biosensor operations [42].

Prostate cancer cell lines LNCaP, LNCaP-GFP and 22Rv1 were cultured in RPMI-1640 (Wisent Bio Products, St-Bruno, QC, Canada) containing 10% fetal bovine serum (FBS) (VWR, Radnor, PA, USA). LAPC4 and LAPC9-GFP cells were cultured in DMEM (Wisent) and IMDM (ThermoFisher Scientific, Waltham, ON, Canada) media, respectively, containing 10% FBS. Production of LNCaP-GFP stably transduced cell lines has been previously described [31]. LNCaP and LAPC9-GFP cell lines were kindly provided by Dr. C. Guillemette and Dr. L. Wu, respectively. Cell lines were tested for mycoplasma using the MycoAlert Mycoplasma Detection kit (Lonza, Basel, Switzerland).

### 2.3. Cell Line Adenoviral Infection and Treatment for Dynamic Bioluminescence Imaging

LNCaP, LNCaP-GFP, LAPC4, LAPC9-GFP and 22Rv1 cells (2000 cells/well) were seeded in a 384-well black plate (Greiner Bio-One North America Inc., Monroe, NC, USA) in RPMI-1640 with 10% charcoal-treated FBS (FBS-CT, Wisent) and dihydrotestosterone (DHT) (Toronto Research Chemicals, Toronto, ON, CA) at 1 nM. The cells were then transduced with either *CMV*-TSTA (1 × 10^4^ infectious viral particle (IVP)) or *PSEBC*-TSTA (1 × 10^5^ IVP) adenoviruses. Bioluminescent imaging was performed as described below by using LV200 bioluminescence microscope. Media were replaced with appropriate treatments (DHT (1 nM), DHT + 10 μM Enzalutamide (Enza, MedChem Express, Monmouth Junction, NJ, USA), DHT + 10 μM Bicalutamide (Bica, Sigma-Aldrich, Oakville, ON, Canada), DHT + 10 μM Apalutamide (Apa, MedChem Express) DHT + 10 μM Darolutamide (Daro, MedChem Express)). Forty-eight hours later, bioluminescence imaging was repeated on the same cells. D-luciferin (3.5 mM) was added 20 min before each imaging time-point.

### 2.4. ECM-M Culture Conditions and Cell Tracking

Here, 48 h after seeding and infection or 24 h after seeding (if infection not needed) in a 384-well black plate, media were partially removed, leaving 10 μL at the bottom of the well. Matrigel^TM^ Matrix High Concentration (HC) or Standard formulation (SF) (Corning, Corning, NY, USA) solution was diluted at different concentrations (10, 20, 40 or 60%) in cold RPMI-1640, with 10% decomplemented FBS charcoal-treated (CT) and DHT (1 nM). Then, 10 μL of this ECM solution was added in each well on the cells. Plates were rapidly centrifuged at 225× *g* for 3 min and incubated for 30 min at 37 °C in 5% CO_2_. Then, 30 µL of RPMI-1640 with 10% FBS-CT and 1 nM DHT was added to each well, and the wells were imaged as described below. After the first imaging, media over ECM-M layer were exchanged to add appropriate treatments and, forty-eight hours after, second imaging was performed. Cells found at the same coordinates in both images were characterized as tracked cells. The number of lost cells was defined as the difference in the number of cells between the first and second imaging. The number of displaced cells was obtained by subtracting the number of tracked cells from the number of total cells in the first imaging.

### 2.5. Viability Assay

LNCaP cells were seeded in a 384-well black plate at a density of 2500 cells/well in RPMI-1640 containing 10% FBS-CT. Twenty-four hours later, a 10 μL layer of 40% ECM-M HC was added in half of the wells, as described above. At time 0, 24 or 48 h, 5 μL of resazurin reagent (300 mg/L) (Cayman Chemical, Ann Arbor, MI, USA) was added to each well and incubated 4 h at 37 °C in a cell culture chamber. Fluorescence of each well was then measured by Fluoroskan Ascent (ThermoFisher Scientific) at an excitation/emission wavelength of 540/595 nm. Cell viability was normalized at 0 h for each condition.

### 2.6. Bioluminescence Microscopy

Bioluminescence imaging was performed using an Olympus LV200 microscope equipped with an EM CCD camera (Andor Ixon 897, Tokyo, Japan. Distibuted by OLYMPUS EUROPA HOLDING GMBH, Hambug, Germany) and an incubation chamber with temperature control, humidity and gas flow for luminescence imaging, transmitted brightfield and transmitted fluorescence imaging. Each luminescence imaging was performed 20 min after adding D-luciferin at 3.5 mM using 40xX objective, with 20 s of exposure per field of view. The emitted photons passed through an open channel without filters and were collected onto an electron-multiplying CCD camera (Andor Ixon 897). For the fluorescence imaging, samples were excited at a wavelength of 470 nm (X-Cite XLED1, Excelitas Technologies, City, MA, USA), and the fluorescence emission of eGFP was collected using the CCD camera. Data analysis and process design for automated image capture were achieved using the cellSens software, V510_UMA_cellSens17 (Olympus, Tokyo, Japan).

### 2.7. Statistical Analysis

Statistical analyses were performed using GraphPad Prism V9 (GraphPad Software, San Diego, CA, USA). Sample unpaired or paired comparisons were analyzed by Mann–Whitney test or Wilcoxon test, respectively. Significance was established at *p*  ≤  0.05 (*), 0.01 (**), 0.001 (***), and 0.0001 (****) levels, as indicated in the figures.

## 3. Results

### 3.1. ECM-M-Based Culture Model Facilitates Cellular Tracking during Dynamic Bioluminescence Imaging and Improves Cell Viability

To obtain optimal expression of the adenoviral system in PCa cell lines and visualize the impact of ARi on the reporter’s expression, cells must be maintained in culture for several days and tracked before and after treatment (48 h). A major limitation of our system is the low traceability of PCa cells throughout the experiment when maintained in tissue culture-treated plates.

**Figure 1:**
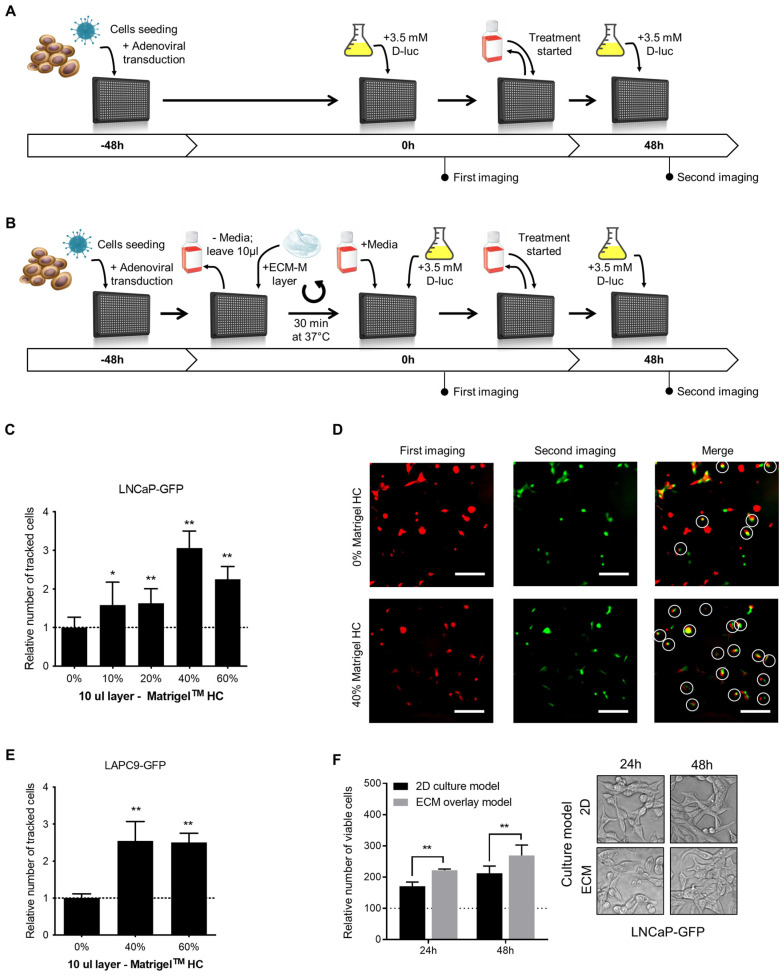
ECM-based culture model facilitates cell tracking in dynamic bioluminescence microscopy (BLM) imaging condition and improve cell viability. (**A**) Schematic representations of the classical 2D single-cell BLM tracking method. (**B**) Schematic representations of the ECM-M modified method. PCa cells were seeded and transduced with specific adenoviral reporter systems in 50 μL of media in a 384-well plate (time -48 h). Forty-eight hours after seeding and transduction (time 0 h), plate was directly imaged (classical method), (**A**) or media were removed before the addition of a diluted solution of ECM-M (ECM-M modified method, (**B**) see M&M for details). After the imaging at time 0, media over cells (**A**) or Matrigel layer (**B**) were changed and at time 48 h, a second imaging was performed in presence of D-luciferin. (**C**) Addition of a 10 μL layer of 40% or 60% Matrigel HC facilitates identification of poorly adherent LNCaP-GFP cells after change of media and 48 h of incubation. (**D**) Between the two imaging, the removal of media and addition of treatment result in a movement of cells. LNCaP-GFP cells were imaged in classical 2D culture condition (0%, before (pseudo-color red) and after (pseudo-color green) change in media and 48 h incubation. By merging the two imaging, only white-circled cells can be tracked. Cells that can be retrieved in the second imaging compared to first imaging were considered as tracked cells (white circles). (**E**) Addition of a 10 μL layer of 40% or 60% Matrigel HC facilitates identification of non-adherent LAPC9-GFP cells after change of media and 48 h of incubation. Relative number of tracked cells was normalized to condition without Matrigel (dotted line). Data represents two distinct experiences in triplicates ± SD. (**F**) In ECM-M culture condition, LNCaP-GFP cells with 40% Matrigel HC slightly increase their viability. No morphological differences can be seen in phase contrast microscopy after 24 or 48 h exposition to Matrigel (right panel). After imaging, cell viability was measured with a resazurin assay. Relative fluorescent unit measurements were normalized to time 0 h of each condition (dotted line). Data represent two distinct experiences in triplicates ± SD (right panel). D-luc: D-luciferin; ECM-M: extracellular matrix (Matrigel), HC: high concentrated. Significance was established at *p* ≤ 0.05 (*), 0.01 (**) levels.

Schematic representations of the classical 2D and proposed ECM-M-based protocols are illustrated in Figure 1A and Figure 1B, respectively. First, we investigated if the ECM-M culture condition would increase the number of traceable poorly adherent PCa cells (LNCaP-GFP), compared to classical 2D culture conditions, in a range of ECM-M concentrations. Ten microliters of 10, 20, 40 and 60% ECM-M High Concentrated (HC) diluted in complete media was tested. To measure the impact of ECM-M addition, cell position in first imaging was compared to that at second imaging (Figure 1C). As shown in Figure 1C,D, a 10 µL layer of 40% and 60% ECM-M HC could increase the number of tracked cells between the two images compared to classical 2D culture conditions by 3.1- and 2.3-fold, respectively. ECM-M Standard formulation (SF) and 5 µL layer of Matrigel HC were also tested, but they did not allow for better stabilization of the cells (Figure S1B,C). Moreover, the removal of media to add ECM-M did not cause significant disturbance of cells (Figure S1D).

To further validate ECM-M culture conditions, we tested a 10 µL layer of 40% and 60% Matrigel HC on a non-adherent LAPC9-GFP prostate cancer cell line. Interestingly, these ECM-M culture conditions could also increase, by 2.54- and 2.51-fold, respectively, the number of tracked cells compared to 2D culture conditions (Figure 1E). To maximize the stabilization effect and to minimize product utilization, we used a 10 µL layer of 40% ECM-M HC. To determine whether LNCaP-GFP cell viability was modulated by ECM-M culture conditions, cells were cultured in the presence or absence of ECM-M, and their viability was quantified using a Resazurin assay. LNCaP cells cultured with ECM-M, showing 1.29-fold (at 24 h) and 1.26-fold (at 48 h) higher viability, respectively, compared to media alone, were quantified based on the number of viable cells in the total well (Figure 1F). We then assessed the effect of ECM-M on cell morphology. LNCaP-GFP cells in ECM-M culture conditions for 24 or 48 h did not show visible changes in their morphology by phase-contrast imaging, displaying a spindle fibroblastic-like shape and limited cell–cell contacts (Figure 1F).

### 3.2. ECM-M-Based Culture Model Is Compatible with Bioluminescence Imaging of PCa Cells

**Figure 2:**
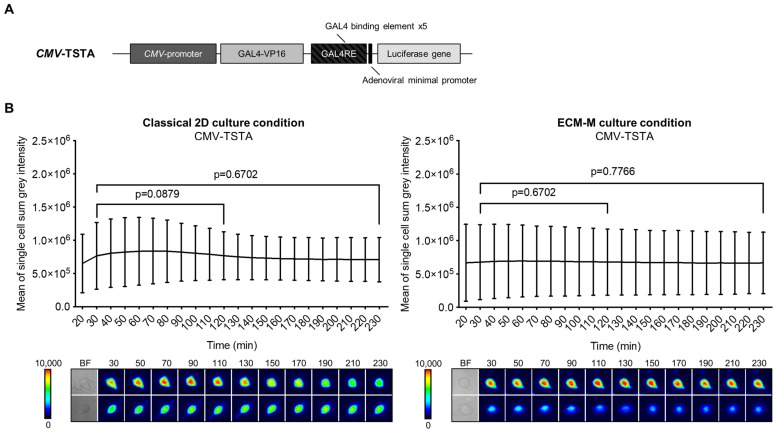
ECM-M culture condition is compatible for imaging prostate cancer cells using bioluminescence imaging method. (**A**) Scheme of the non-replicative *CMV*-TSTA adenovirus with the ubiquitous CMV promoter. (**B**) LNCaP-GFP cells were seeded and infected with 1 × 10^4^ IVP of *CMV*-TSTA adenovirus. Seventy-two hours later, a 10 μL layer of 40% Matrigel was added in half of the wells as described previously. After adding D-luciferin (3.5 mM), images were taken every 10 min during 230 min. Cell bioluminescence activities were assayed by means of the cellSens V510_UMA_cellSens17 software. Mean sum gray intensity/cell was obtained from 10 randomly selected cells by replicate. Data represent technical triplicates ± SD. Wilcoxon paired test was used. Lower panels show 2 representative low and high single-cell luminescence signals over time. The corresponding bright field (BF) imaging of the cells tracked is all also shown. ECM-M: extracellular matrix (Matrigel).

Our technology involves molecular imaging based on bioluminescence signal emission. To ensure that our ECM-M model was permeable to D-luciferin (substrate of Firefly luciferase (Fluc) protein), we compared the bioluminescent signal produced by LNCaP cells infected by CMV-TSTA, an adenovirus expressing Fluc under the control of the ubiquitous CMV promoter (Figure 2A), with or without ECM-M. In both culture conditions, Fluc activity was easily detectable for single-cell imaging after 30 min of D-luciferin addition. Moreover, we detected a sustained Fluc signal, under both conditions, with no significant reduction in intensity from 30 min to 230 min following substrate exposure (Figure 2B). These results indicate that the ECM-M layer does not interfere with bioluminescence microscopy efficacy and enables dynamic bioluminescence studies.

### 3.3. ECM-M Culture Model Supports Bioluminescence Microscopy Accuracy to Measure Androgen Receptor Activity Modulation by Antiandrogens

**Figure 3:**
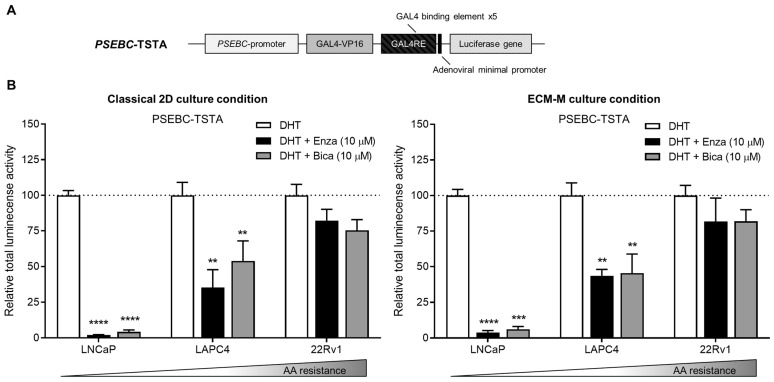
Extracellular matrix culture condition allows bioluminescence microscopy accuracy to measure androgen receptor activity modulation by antiandrogens. (**A**) Scheme of the non-replicative *PSEBC*-TSTA adenovirus with the androgen responsive PSEBC promoter. (**B**) Graphs show response to antiandrogen treatment detected with *PSEBC*-TSTA. Prostate cancer cells (LNCaP, LAPC4 or 22Rv1 cells) were infected with 10^5^ virus particles of *PSEBC*-TSTA in 384-well plate. Seventy-two hours after infection, ECM-M overlays were added or not on the cells and treatments were added (DHT (1 nM), DHT (1 nM) + Bica (10 μM) or DHT (1 nM) + Enza (10 μM)). Forty-eight hours post-treatment, imaging using the bioluminescence microscope was done to assess response to AA by determining the total luminescence activity of positive cells per well. Relative total luminescence activity was normalized to condition with DHT (dotted line). ** *p* < 0.01, 0.001 (***), **** *p* < 0.0001. AA: antiandrogen; Bica: bicalutamide; DHT; dihydrotestosterone; ECM-M: extracellular matrix (Matrigel); Enza: enzalutamide. Data represent one experiment in triplicates ± SD.

To ensure that ECM-M culture conditions did not interfere with the detection of AR transcriptional activity using our biosensor, we tested if *PSEBC*-TSTA (Figure 3A) could still monitor bulk cell line sensitivities to ARi in our model. PCa Ari-sensitive (LNCaP, LAPC4) and -resistant (22Rv1) cells were infected with *PSEBC*-TSTA and exposed to the following treatments: DHT (AR agonist (1 nM)), DHT + Enza (10 µM) or DHT + Bica (10 µM) [42,43]. Total luminescence activity was measured for each treatment group and was normalized to the DHT-only treated group (Figure 3B). Under DHT + Enza (10 µM) and DHT + Bica (10 µM) treatments in 2D classical culture conditions, the total luminescence activity decreased to 2.0% and 4.3% in LNCaP and to 35.4% and 53.9% in LAPC4, respectively, when compared to DHT alone. In ECM-M culture conditions, total luminescence activity decreased to 3.7% and 6.2% in LNCaP and 43.6% and 45.5% in LAPC4, under Enza and Bica treatment, respectively, in comparison to DHT alone. In the AA-resistant 22Rv1 cell line, the AR luminescence activity signal in both classical 2D and ECM culture conditions remained high under Enza (82.2% and 81.7%, respectively) or Bica (75.4% and 81.8% of DHT group, respectively) treatments (Figure 3B). Overall, these results show that the ECM-M culture condition is, therefore, suitable to monitor AR activity and, thus, ARi sensitivity in PCa cell lines.

### 3.4. ECM-M-Based Culture Model Allows for Accurate Tracking of Each Single Cell to Determine its Sensitivity to ARi by Bioluminescence Microscopy

**Figure 4:**
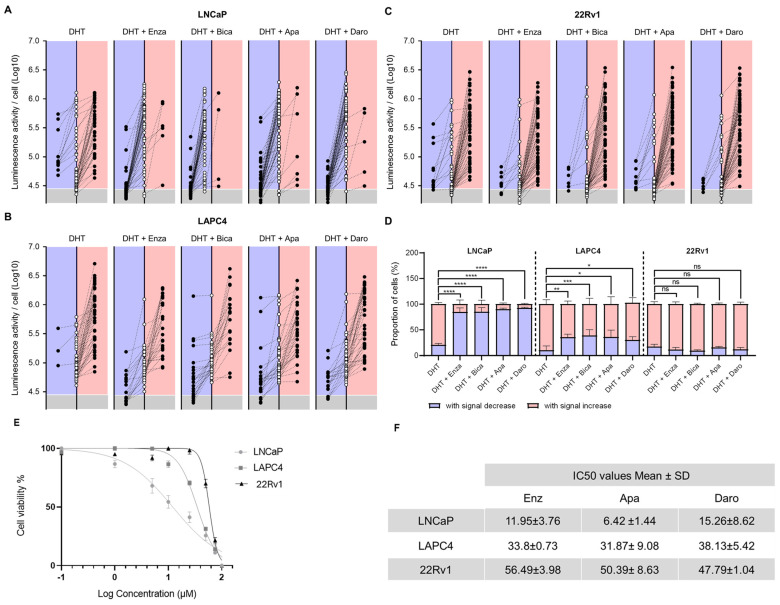
ECM-M-based ECM culture model allows for accurate tracking of each single-cell AA sensitivity by bioluminescence microscopy. Randomly selected LNCaP (**A**), LAPC4 (**B**) and 22Rv1 (**C**) cells infect with PSEBC-TSTA from extracellular matrix (ECM) overlay culture condition of a representative experiment were tracked between baseline and after treatment imaging to determine their change in androgen receptor (AR) activity. Bioluminescence status of each cell over time is represented as an empty circle (baseline) connected to a full circle (after treatment). Forty-eight hours bioluminescence statuses (full circles) of cells that have an increase in luminescence activity are illustrated in right red panels and those with a decrease in Fluc expression are illustrated in left blue panels. Bioluminescence statuses (circles) place in the gray area are under the limit of detection. Luminescent cells were counted by means of the cellSens software. (**D**) Proportions of cell signal profiles in each cell lines under DHT control or treatment (DHT + Enza (10 μM), DHT + Bica (10 μM), DHT + Apa (10 μM), and DHT + Daro (10 μM)) conditions. Data represent mean ± SD of three independent experiments. (**E**) Cell viability assay for PCa cell line treated with drugs Enzalutamide. (**F**) IC50 values of three antiandrogen drugs (Enzalutamide, Darolutamide and Apalutamide). Data are presented as the mean ± standard deviation. PCa cell lines (LNCaP, LAPC4 and 22Rv1) were treated with antiandrogen drugs (Enz, Dar and Apa) at various concentrations for 120 h, and cell viability was determined using a colorimetric Resazurin assay. Apa: apalutamide; Daro: darolutamide; DHT: dihydrotestosterone; Bica: bicalutamide; Enza: enzalutamide, Fluc: Firefly luciferase. Significance was established at *p*  ≤  0.05 (*), 0.01 (**), 0.001 (***), and 0.0001 (****) levels.

Cell culture manipulations in the original 2D dynamic imaging protocol (Figure 1A) were difficult due to single-cell loss and tracking uncertainty. The ECM-M culture condition decreases the mobility of cells and, thus, facilitates their tracking between two consecutive imaging sessions (Figure 1D). To investigate the enhanced capability of the method, PCa cell lines were seeded, infected with *PSEBC*-ITSTA and imaged before and after treatment with DHT or androgen inhibitors. By determining the initial and final bioluminescence status of each cell, we could visualize the impact of treatment on AR activity at the single-cell level. In Figure 4A–C, randomly selected single cells from ECM-M culture conditions were tracked between first and second imaging to determine their change in Fluc expression after treatment exposure. The bioluminescence signal associated with each cell over time is represented as an empty circle (baseline status) connected to a full circle (after treatment status). The final bioluminescence statuses (full circles) of single cells that have an increase in Fluc expression are illustrated in the right red panels, and those with a decrease in Fluc expression are illustrated in left blue panels. Bioluminescence statuses (circles) placed in the gray area are under the limit of detection.

In LNCaP cells treated with the AR agonist DHT, 77.7% of cells exhibited increased Fluc activity, while 22.3% showed a decreased signal. When exposed to DHT + Enza, only 15.3% of cells displayed increased luminescence, while 84.7% showed decreased Fluc activity, highlighting an important ARi-responsive cell subpopulation (Figure 4D). Of these, 48.7% showed no detectable luminescence signal following treatment. Similar trends were seen with other FDA-approved ARi, Apa and Daro, with 90.2% and 92.7% of cells showing decreased luminescence (Figure 4A,D). Almost all LAPC4 cells treated with DHT increased their Fluc activity (91.2%), while treatment with Enza, Apa or Daro induced a significant decrease in this category of cells to 35.4%, 35,8% and 30%, respectively (Figure 4B,D). For 22Rv1 cells, no significant difference was observed in luciferase activity between DHT- and ARi-treated groups (Figure 4C,D), pointing to the presence of a large ARi non-responsive subpopulation in this cell line. Notably, no significant differences among ARi treatments were observed across all tested cell lines.

To link the antiandrogen responses of each cell line tested to their respective drug growth inhibition, we established a dose–response survival curve for each ARi and calculated the cytotoxic concentration at which 50% of cells were viable (IC50) (Figure 4E). The overall response of the cell lines evaluated with the bioluminescence assay was in accordance with their sensitivity to the different drugs in the cell-based cytotoxicity assay. LNCaP, which had the lowest IC50 for all drugs, had the most cells decreasing their AR activity upon ARi treatment. 22Rv1 cells, on the other hand, displayed the highest IC50 and had a small proportion of cells affected by drug treatment in our Fluc assay.

These results demonstrate that our ECM-M-based culture model, in combination with the PSEBC-TSTA biosensor and bioluminescence microscopy quantitative capabilities, allows for the characterization of cell lines’ ARi responsiveness at a single-cell level within a heterogeneous cell population. Importantly, our findings reveal a direct correlation between the IC50 value and the proportion of non-responsive cells. As the IC50 value increases, we observe a corresponding rise in the number of cells within the population that do not respond to androgen receptor inhibition.

## 4. Discussion

The quantification of heterogeneous antiandrogen responses within cell populations is critical for advancing the management of PCa. However, capturing dynamic single-cell drug responses has remained a technical challenge. This study aimed to enhance the culture conditions of PCa cells to enable the monitoring of single-cell AR activity when using biosensors such as PSEBC-TSTA and bioluminescence microscopy. We show that the addition of an ECM-M layer to PCa cells improved cell tracking over time without interfering with bioluminescence imaging. The ECM-M coating demonstrated no hindrance to substrate permeation nor signal output. Moreover, it preserved AR responsiveness when compared to culture conditions without ECM-M. Importantly, this method facilitated the single-cell tracking of AR responses to antiandrogens during dynamic imaging, allowing for the characterization of the cell-by-cell heterogeneity of anti-androgen responses within different PCa cell populations.

Through single-cell bioluminescence imaging, we successfully identified subpopulations within bulk LNCaP and LAPC4 cells that exhibited varying responses to antiandrogens. Surprisingly, even within the generally sensitive cell populations, there were instances where cells showed limited sensitivity to androgen receptor inhibition. Specifically, when compared to the DHT condition, we observed the presence of cells in the LNCaP and LAPC4 populations that did not display a statistically significant reduction in signal under anti-androgen treatment. This intriguing observation highlights the capability of our method to discern a subpopulation of cells that experienced a minimal loss of androgen response, potentially indicating an inherent resistance or a secondary effect to the treatment within an otherwise sensitive cell population. Moreover, within the ECM environment, cells that exhibited no detectable signals in one set of images could still be located and included in the single-cell analysis through complementary imaging using phase-contrast observation. This aspect is particularly valuable, especially in situations where the quality of brightfield contrast is suboptimal, as encountered under our specific Olympus LV200 imaging conditions.

ECM-M is minimally processed, and it provides a good replicate of an in vivo ECM [44,45]. It can form a transparent and permeable gel at 37 °C, making it ideal for tracking single cells over time using bioluminescence microscopy [38]. Moreover, ECM components regulate cell proliferation, differentiation, migration, survival, adhesion, as well as cytoskeletal organization and cell signaling in normal and diseased states, such as cancer [46,47]. Thus, it is not surprising that the composition of the ECM-M along with its physical properties can also influence the cell response to drugs by either enhancing drug efficacy, altering a drug’s mechanism of action or by promoting drug resistance. For example, Stock et al. compared the drug response profiles of PCa cell lines to Enza and revealed that the maximal growth inhibitory effect was reduced to about 30–40% when cells were grown in ECM-M-based embedded culture systems compared to 2D cultures [48]. Thereby, compared to the classical 2D dynamic imaging method, our ECM-M cell culture model could better mimic in vivo systems, being a promising and more reliable tool for testing drug heterogeneous responses. However, because ECM-M is processed from natural sources, batch-to-batch variability may be seen in the purified scaffold. Also, Matrigel is available in liquid form and requires handling at cold temperatures to avoid premature gelation. Consequently, the use of synthetic replacement matrices in future studies might provide the means to disentangle the matrix from component-dependent effects and from the requirement to work at low temperatures.

In addition to mutational polyclonality, single cells within a tumour will intrinsically vary in the activity of their signaling, influencing the biological properties and therapeutic vulnerabilities of distinct tumour cell subpopulations [49,50]. However, the impact on intra-tumoral polyclonality and heterogeneity on treatment responsiveness and/or resistance are not currently well understood. By capturing the heterogeneity of AA responses at a single-cell level, our ECM bioluminescence imaging method represents an interesting tool to link single-cell drug responses to intra-signalling modulation. In the last few years, the technological advances in DNA sequencing tools have exposed the whole genome, the exome and the transcriptome for single-cell analysis [51,52,53]. Coupling single-cell drug responses and single-cell molecular characterization technologies may provide a better understanding of how PCa cells respond heterogeneously to AA and reveal characteristics of subpopulations resistant to this treatment.

Furthermore, the ability to perform single-cell analysis in the context of the antiandrogen (ARAT) response offers a promising avenue for addressing the complex issue of resistance and optimizing treatment choices. This study provides evidence that the PCa cell response to ARATs is highly heterogeneous, with distinct subpopulations exhibiting varying degrees of sensitivity or resistance. By capturing this heterogeneity at the single-cell level, our method allows for the identification of drug-sensitive subpopulations within sensitive cell line populations, which could have implications for treatment selection. For instance, a patient with a response profile like LNCaP cells might be a better candidate for androgen deprivation therapy (ADT) alone, whereas a patient with a response profile like LAPC4 cells might benefit from a combination therapy of ADT, chemotherapy and ARPi [54]. The dynamic imaging of single-cell AR activity under ARPi exposure facilitates the evaluation of the proportion of cells that lose androgen response, either non-specifically or due to treatment. In addition, this approach avoids the oversimplification of treatment choices based on bulk tissue biomarkers, which can lead to the exclusion of potentially effective therapies or the inclusion of therapies that have minimal impacts on the disease.

In conclusion, our study introduced a robust protocol for ECM overlay culture dynamic bioluminescence imaging, demonstrating its efficacy across various prostate cancer (PCa) cell lines. The ECM-M-based culture model facilitated the identification of heterogeneous antiandrogen response subpopulations at the single-cell level, underscoring its utility in studying drug responsiveness within complex cell populations. Through the utilization of single-cell isolation methods like the Qiascout instrument, we successfully isolated responder and non-responder subpopulations within the same cell population. This achievement marks a pioneering step in the separation of such subpopulations, representing a novel advancement in our understanding of antiandrogen response heterogeneity. Having achieved success in this crucial step, our next focus is on exploring the transcriptional activity of transcription factors in response to antiandrogen treatments using single-cell transcriptomics approaches. This avenue of investigation is pivotal for understanding the molecular basis of cellular heterogeneity, drug resistance mechanisms and their implications for treatment outcomes in PCa.

## Data Availability

All data are available from authors upon request.

## Supplementary Materials

The following supporting information can be downloaded at: https://www.mdpi.com/article/10.3390/bios14040175/s1, Figure S1: Additional ECM-M condition characterization.
