# Supplementary material for: An Extracellular Matrix Overlay Model for Bioluminescence Microscopy to Measure Single-Cell Heterogeneous Responses to Antiandrogens in Prostate Cancer Cells
Source: Biosensors (Basel). 2024 Apr 5;14(4):175. doi: 10.3390/bios14040175 (PMC11048191; doi:10.3390/bios14040175)
Supplement: Supplementary file 1 [file biosensors-14-00175-s001.zip › biosensors-2893179-supplementary.pdf]

Supplementary figure

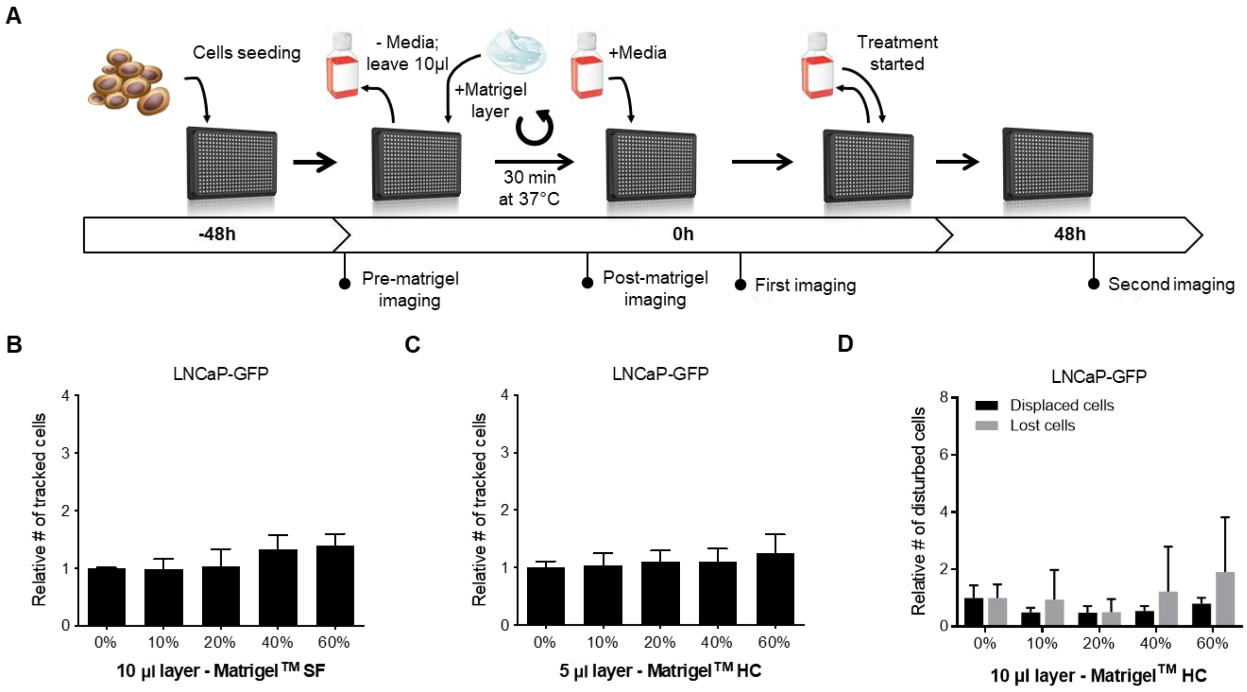

Figure S1: Additional ECM-M condition characterization.

(**A**) Schematic representations of the ECM-M characterization protocol of dynamic bioluminescence imaging. PCa cells were seeded in 50 µl of media in a 384-well plate (time -48 h). Forty-eight hours after seeding, a pre-Matrigel on-top coating imaging was done, media was removed to leave 10 µl at the bottom of the wells, diluted solution of Matrigel was added on the cells, plate was centrifuged and incubated for 30 min at 37 °C, and a post-Matrigel imaging was done. Then, media was added to reach a total volume of 50 µl and the wells were reimaged (First imaging). After this imaging, media over Matrigel layer was changed to add appropriate treatment and forty-eight hours after, another imaging (Second imaging) was performed. (**B-C**) Matrigel Standard formulation (SF) (**B**) and 5µl layer of Matrigel high concentrated (HC) (**C**) did not allow a better stabilization of the cells between first and second imaging. Cells that can be retrieved in the second imaging compared to first imaging were considered as tracked cells. Relative number of tracked cells was normalized to condition without Matrigel. (**D**) Removal of the media to further add Matrigel HC does not cause significant loss of cells. Cells that can be retrieved after Matrigel addition (post-Matrigel imaging) compared to the imaging before removing the media (pre-Matrigel imaging) were considered as tracked cells. The number of cells in the post-Matrigel imaging was subtracted to the number of cells in the pre-Matrigel imaging to determine the difference between the two imaging and this value was considered as the number of lost cells from ECM addition (lost cells = total cells in pre-Matrigel imaging – total cells in post-Matrigel imaging). The number of tracked cells in two imaging was subtracted to the number of cells in the first imaging to determine the numbers of displaced cells by ECM addition (displaced cells = total cells in pre-Matrigel imaging – number of tracked cells (pre vs post-Matrigel imaging)). Data represent two distinct experiences in triplicates ± SD.
